# Effect of visual attention on the properties of optokinetic nystagmus

**DOI:** 10.1371/journal.pone.0175453

**Published:** 2017-04-07

**Authors:** Kei Kanari, Kiyomi Sakamoto, Hirohiko Kaneko

**Affiliations:** 1 Department of Information and Communications Engineering, School of Engineering, Tokyo Institute of Technology, Yokohama, Kanagawa, Japan; 2 Groupwide CTO Office, Technical Information Service Department, Panasonic Corporation, Kadoma, Osaka, Japan; University of Muenster, GERMANY

## Abstract

It has been demonstrated that optokinetic nystagmus (OKN) gain increases through attention to peripheral motion when the central visual field is occluded. However, how the properties of OKN change when two areas containing motion in different directions are presented in the peripheral visual field is still unclear. In this study, we investigated whether OKN corresponding to the attended motion in the periphery occurred while the observer was maintaining fixation at the center. We presented two areas with different directions of motion arranged on the left and right, top and bottom, or center and surrounding (concentric) areas in the display. Observers counted targets appearing on the attended area in the stimulus to maintain their attention on it. The results indicate that attention enhances the gain and frequency of OKN corresponding to the attended motion even in the case of stimuli having several areas with different directions of motion.

## Introduction

To receive visual inputs appropriately, our eyes attempt to keep the image of an object at the same location on the retina using voluntary and involuntary eye movements. Smooth pursuit is a voluntary eye movement to keep the image of a moving object on the fovea up to a target velocity of about 60 deg/s. Vestibulo-ocular reflex (VOR) is an involuntary eye movement, produced by the rotation and translation of the head and body in space to reduce retinal slip of the image. Optokinetic nystagmus (OKN) is also an involuntary eye movement and a rhythmic oscillation of the eye produced by a moving visual field [1].

OKN consists of a slow phase (pursuit movements in the direction of stimulus motion) and a fast phase (saccadic return movements opposite to the direction of motion); it has characteristics related to the stimulus’ physical features. It is generally known that the gain of OKN (the ratio of the slow phase velocity to stimulus velocity) decreases when there is a stationary object in the plane of the moving stimulus and greatly decreases if edges perpendicular to the direction of stimulus motion are presented [2]. It is also known that OKN gain decreases as the width or area of the moving stimulus decreases [3]. Some studies have reported that OKN gain decreases when the central visual field is occluded [4–7] but one of the studies has shown that the rise in time of OKN gain was similar for the central and peripheral stimuli indicating that the mechanisms for central and peripheral stimuli are basically the same [8]. In addition, OKN is almost completely suppressed when the central visual field is occluded and a stationary edge perpendicular to motion is presented [9].

OKN is influenced by visual attention [10–12], as well as stimulus features. For example, OKN gain decreases when the central visual field of motion stimulus is occluded; however, OKN evoked by the motion stimulus in the peripheral visual field can be facilitated by attention paid to the motion [5,6]. OKN is elicited by the motion focused on by the observer when two moving patterns in different directions are superimposed on the same depth plane [13].

However, the relative effects of visual attention, retinal position of motion, and other factors on OKN have not been shown clearly. All of the previous studies [5,6,11,12] except one [14] mentioned below have investigated the properties of OKN only when the unidirectional motion was presented in the whole visual field. A previous report stated that OKN is not produced by peripheral motion when a motion having a different direction was presented at the center, even if observers were attending to the peripheral motion [14]. From this result, the visual system seems to prefer motion at the fovea than at the periphery even if it is attended for the occurrence of OKN. However, as stated by Howard and Gonzalez, the binocular fusion of the motion image, and not attention to the motion, might be the important factor. But, in their study to present two areas having different directions of motion, attention was not manipulated precisely. Observers were simply instructed to attend to the motion stimulus and were not given an attentional task. In addition, visual attention can be classified into at least two types−exogenous (a stimulus-driven process) and endogenous (a volitional process) attention [15]; and the effects of the different kinds of attention have not been shown clearly. Therefore, it is still unclear how OKN relates to spatial attention paid to an area in the stimulus with various directions of motion.

This study aimed to investigate how motion information on the fovea and that in an attended area interacted to produce OKN when several areas containing motion with different directions were presented. Visual attention can be redirected to one location while the line of sight is maintained on another location. In such a situation, our visual system should allow an observer to stabilize the images of the attended motion and prevent OKN from being driven by motion from another location, for example, while walking forward. But we don’t know the details of such aspects of the visual system.

For this purpose, we examined whether OKN corresponding to an attended motion occurred and how OKN was modulated by attention when two areas having different directions of motion with various layouts were presented while manipulating the state of attention with an attentional task and maintaining fixation at the center. In addition, we mention a way of estimating attentional location and state from OKN and motion in the visual scene from the relationship between OKN and attention obtained in this study.

## Experiment 1

In Experiment 1, we investigated whether OKN corresponding to the motion direction of attended stimulus occurred when two areas with different motion directions were presented at symmetrical locations around the fixation. The motion stimuli were presented at the top and bottom or left and right on the display. Observers were instructed to attend to one of the motion stimuli, indicated by a visual cue, while fixating at the central line. Observer attention was kept constant by a task of counting targets distributed in the motion area. Eye movements were measured during the trial and analyzed to examine how attention modulated the gain and frequency of OKN.

### Methods

#### Stimuli

**Fig 1:**
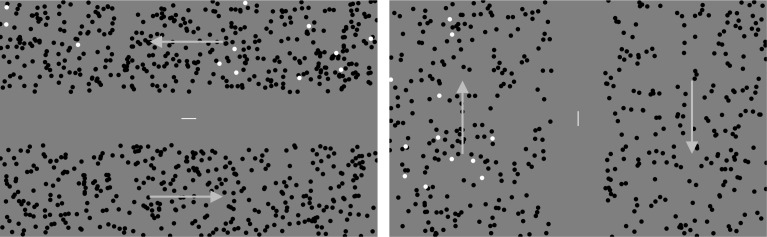
Stimulus configuration used in Experiment 1. Left and right panels, respectively, show LR and UD patterns. White dots indicate the targets. The line at the center shows the fixation line. Arrows show the directions of motion.

The motion stimulus consisted of randomly distributed dots. The size, luminance, and velocity of an individual dot was 0.66 deg, 0.12 cd/m^2^, and 13.3 deg/s, respectively. Dot density in the stimulus was 1.6 dot/deg^2^. The luminance of the background was 25.0 cd/m^2^. In the LR (left/right) pattern, stimuli (34.1 × 10.2 deg) having leftward and rightward motions were simultaneously presented at the top and bottom of the fixation (Fig 1, left panel). Moreover, in the UD (up/down) pattern, stimuli (13.0 × 28.4 deg) having upward and downward motions were simultaneously presented to the left and the right of the fixation (Fig 1, right panel). Two motion stimuli were separated by 8 deg. The observer maintained attention on the area of motion with a task of counting targets distributed in the area. The size and velocity of the target dot were the same as those of the other dots in the motion stimulus. The luminance and density of the target dot were 92.6 cd/m^2^ and 0.16 dot/deg^2^, respectively. To keep the observers’ eyes on the center of the display screen while not inhibiting OKN parallel to the direction of motion, a fixation line (length 1.1 deg; width 0.16 deg; luminance 31.4 cd/m^2^) was presented at the center. The line was horizontal in the LR pattern and vertical in the UD pattern.

#### Procedure

**Fig 2:**
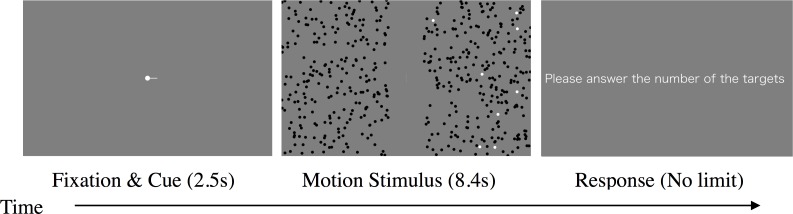
Time course of stimulus presentation in Experiment 1. Observers fixated at the screen center and counted targets (white dots) in the area of attention indicated by the cue.

In one trial, first, a fixation point and a cue to indicate stimulus to attend to were simultaneously presented for 2.5 s, following the signal of pressing a button by the observer. The attentional cue was a line extended to the top or bottom from the fixation point in the LR pattern and to the left or right from the fixation point in the UD condition (Fig 2 left). Motion stimuli were then presented for 8.4 s (Fig 2 center). During the presentation, observers attended to one of the motion stimuli and counted the targets of white dots in the stimulus while fixating on the central line. Targets were always presented in the area indicated by the attentional cue. Each target always appeared at the origin of the motion in the stimulus, moved continually to the other side, and disappeared. Observers responded with the number of targets by using a keyboard after the presentation of motion stimuli (Fig 2 right). The response time was unrestricted and feedback was not provided to the observer. The next trial was launched by the signal of pressing a button by the observer. The location to attend to and the combination of the direction of motion were randomized for each trial. The LR and UD patterns were conducted in separate sessions. There were four conditions of direction of motion to attend to (left, right, up, and down), and the trial of each attended direction was repeated five times for each observer such that we ran a total of 20 trials for each observer. We call this experimental condition the “attention condition.”

As a control, an identical procedure was repeated for an identical stimulus without the attention task. We call these trials the “no-attention condition.”

As another control, we measured OKN when observers attended to the motion stimulus with direct viewing. In this “direct condition,” the same stimulus used in the attention condition was presented at the center of the display. The size of the motion stimulus was 34.1 × 10.2 deg when the motion direction was leftward or rightward and 13.0 × 28.4 deg when the motion direction was upward or downward; the fixation line was not presented. The direction of the stimulus motion was one-way over the whole display. As in the attention condition, there were four conditions of motion direction (left, right, up, and down), and each direction was repeated five times for each observer.

#### Apparatus and observers

The observers sat in a dark room with their head fixed by a chin rest and a bite bar, viewing a CRT monitor (GDM F500R, SONY, 2048 × 1536 pixels, 35.0 × 28.8 cm, 34.1 × 28.4 deg) from a distance of 57 cm. The stimulus was produced and presented using a PC (MacBook Pro, Apple) with MATLAB (MathWorks) and Psychophysics Toolbox extensions [16–18]. The observers responded using a numeric keyboard.

The right eye was recorded with an EyeLink CL (SR Research), a video-based eye tracker, sampling data with 1000 Hz. The data for 200 ms around the eye blinks were excluded from the analysis. We defined OKN as a series of eye movements that had a slow phase and a following fast phase. The fast phase was detected using an eye velocity threshold of 20 deg/s. A slow phase velocity was calculated by averaging velocities for 50 ms just before each fast phase in each trial. Then the gains were averaged over five repetitive trials under each condition for each observer. The gain of each OKN was defined as a ratio of slow phase velocity to the stimulus velocity (13.3 deg/s); it was defined as zero when the frequency of OKN for the trial was zero.

One author and ten naïve volunteers (eight males and three females, aged between 22 and 32 years) participated in this experiment. All had normal or corrected-to-normal visual acuity. Informed consent (written) was obtained from each observer before the experiment. This study was approved by the Tokyo Institute of Technology Epidemiological Research Ethics Committee and carried out in accordance with the Code of Ethics of the World Medical Association (Declaration of Helsinki).

### Results

**Fig 3:**
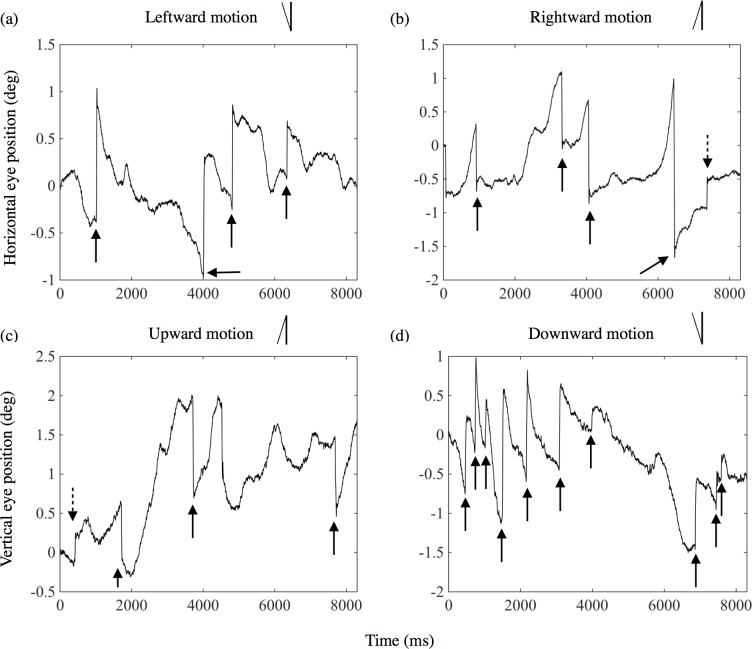
Representative trace of eye position in each trial for an observer (OB1) in Experiment 1. The four panels depict the results when attending to (a) leftward, (b) rightward, (c) upward, and (d) downward motions. The line inserted in the upper part of each panel presents the OKN corresponding to the direction of attended motion. Solid line arrows show the point of OKN corresponding to the direction of attended motion. The dotted line arrow in (b) shows the point of OKN not corresponding to the direction of attended motion.

A representative tracing of eye position for one observer (OB1) during one trial is shown in Fig 3. Each panel presents the result when attending to leftward (a), rightward (b), upward (c), or downward (d) motion, respectively. The horizontal axis presents the time [ms] from stimulus onset. The vertical axis presents the horizontal position of the eye in the LR pattern ((a) and (b)), signed positive when it is in the right. In the UD pattern ((c) and (d)), the vertical axis presents the vertical position of the eye, signed positive when it is in the upper side of the display. The line drawing inserted in the upper part of each panel presents the shape of the predicted OKN corresponding to the direction of the attended motion. For example, in the case when observers attended to leftward motion (Fig 3A), the position of the eye is predicted to move slowly to the left on the display to pursue the motion and then move quickly back to the right.

In Fig 3A, OKN corresponding to leftward motion can be observed around 1000, 4000, 5000, and 6200 ms from the stimulus onset (indicated by arrows). Similarly, in Fig 3B, OKN corresponding to rightward motion occurred around 1000, 3200, 4000, and 6200 ms, although OKN not corresponding (opposite) to the rightward motion can be seen once around 7200 ms (indicated by an arrow with a dotted line). In Fig 3C, OKN corresponding to the upward motion occurred three times, although OKN opposite to the upward motion can be seen once around 500 ms. In Fig 3D, OKN corresponding to downward motion also occurred ten times. No OKN not corresponding to stimulus motion can be seen in the trials.

**Fig 4:**
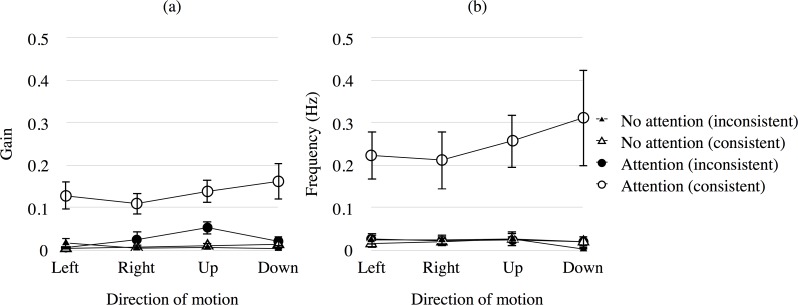
Mean gain and frequency of OKN in Experiment 1. (a) Mean gain and (b) frequency of OKN with consistent and inconsistent directions to the attended stimulus during the attention and no-attention conditions in Experiment 1. The open and filled symbols show the result of consistent OKN (corresponding to the motion direction of attention area containing targets) and inconsistent OKN (opposite to the motion direction of the white targets), respectively. Error bars show ±1 SEM.

Fig 4 presents the mean gain (a) and frequency (b) of OKN for each combination of attention condition and direction of attended motion across observers. The x-axis shows the direction of motion. The y-axis shows gain (a) and frequency (b) of OKN. The circle and triangle symbols show the results of the attention condition and those of the no-attention condition, respectively. The open and filled symbols show the results of consistent OKN (corresponding to the motion direction of attention area containing the white targets) and those of inconsistent OKN (opposite to the motion direction of the white targets), respectively. Error bars show ±1 SEM. From the results, it is obvious that OKN consistent with the direction of the attended motion occurred regardless of the direction of motion and that few OKN occurs when observers did not attend to the motion stimulus. In addition, the gain of consistent OKN in the attention condition was larger than that of inconsistent OKN and OKN in the no-attention condition.

**Fig 5:**
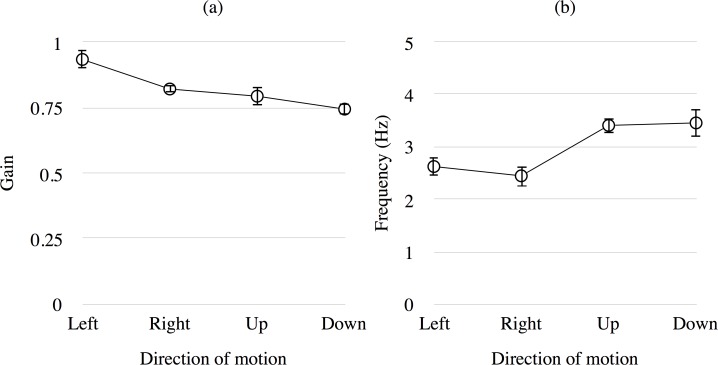
Mean gain and frequency of OKN in the direct condition of Experiment 1. The y-axis indicates the gain (a) and frequency (b) of OKN. Error bars show ±1 SEM.

Fig 5 presents the mean gain (a) and frequency (b) of OKN in the direct condition. The x-axis indicates the direction of motion, while the y-axis indicates the gain (a) and frequency (b) of OKN. Error bars show ±1 SEM.Comparing the results in Figs 4 and 5, it is obvious that more OKN generally occurred in the direct condition than in the attention condition. This result confirms that the stimuli projected on a flat screen used in this study produces a similar OKN as for the general stimulus used to generate OKN for the stimulus projected on a hemi-field screen or a rotating drum. In addition, there was a directional dependency for the frequency of OKN in the direct condition, though there was no directional dependency for the gain. To test the effect of the direction of motion on the gain and frequency of OKN, a one-way ANOVA was performed on the result in the direct condition. The main effect of the *direction of motion* was significant for the frequency of OKN (*F*(3, 30) = 11.967, *p* < .001). Multiple comparison tests using Ryan’s method (α = 0.05) showed that the difference in orthogonal motion conditions differed significantly (*p* < .05). The main effect of the *direction of motion* was not significant for gain of OKN (*F*(3, 30) = 1.199, *p* > .10). At this point, the reason for the inconsistency in the results of frequency and gain is not known.

To confirm that observers did not directly look at the motion stimulus during the trials, we precisely analyzed eye position. The average and standard deviation of eye position were calculated for each trial after the subtraction of the baseline, which was the average data during the 50 ms before the onset of motion stimulus. The average (and standard deviations) of horizontal position for vertical motion were 0.51 (0.42), signed positive if it was to the right direction. Moreover, that of vertical position for horizontal motion were 0.001 (0.06), signed positive if it was to the upper direction. These results demonstrate that the observers did not look directly at the motion stimulus because the edge of the motion stimulus was 4 deg apart from the fixation line. Therefore, we conclude that OKN in this experiment did not occur by looking directly at the motion stimulus but through visual attention.

## Experiment 2

A previous study stated that OKN corresponding to the attended motion in the peripheral visual field did not occur when stimuli with different directions were presented at the central visual field [14]. We supposed, however, that one of reasons for this result was due to the shortage of attention to the peripheral stimulus, as there was no task to draw attention to in the experiment. In Experiment 2, we investigated whether OKN corresponding to the attended motion in the periphery occurred even when stimulus moving to the opposite direction was presented in the central visual field, while manipulating the state of attention using an attentional task.

### Methods

#### Stimuli

**Fig 6:**
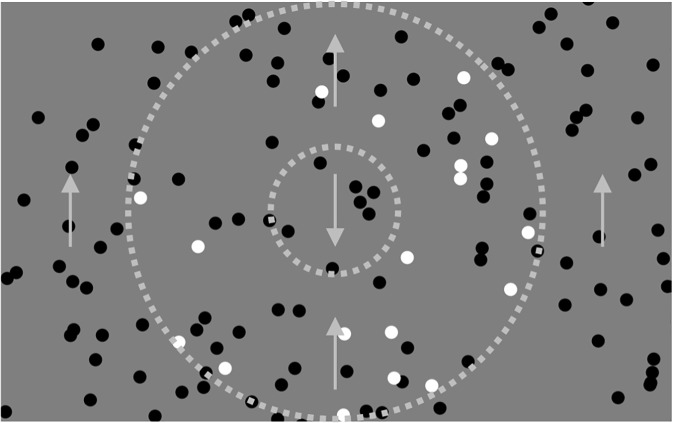
Stimulus configuration used in Experiment 2. White dots show targets. Arrows show direction of motion. Circles with dotted lines (not presented actually) show borders of areas where targets were presented.

The motion stimulus consisted of randomly positioned moving dots. The size, luminance, velocity, and density of a dot were 1.1 deg, 0.12 cd/m^2^, 13.3 deg/s, and 1.6 dot/deg^2^, respectively. The luminance of the background was 25.0 cd/m^2^. The central stimulus was circular, with a diameter of 9.4 deg, and presented at the center of the display (the inner circle with dotted line in Fig 6). The peripheral stimulus having an opposite motion to the central motion was presented in the rest of the display. Target dots used for the attentional task (see the next section for details) were presented in the annular area outside of the central stimulus, shown as white dots in an area surrounded with two concentric dotted lines in Fig 6. The dotted lines were not actually presented. The size and velocity of the target dot were the same as those of other dots in the motion stimulus. The luminance and density of the target dot were 92.6 cd/m^2^ and 0.4 dot/deg^2^, respectively. The fixation line was presented in the center in the same way as in Experiment 1.

#### Procedure

In this experiment, there were two conditions of attention: “peripheral attention,” and “central attention.” In the “peripheral attention” condition, observers were instructed to attend to the peripheral stimulus before each trial was launched. After the observer pressed the button, the fixation point was presented for 2.5 s. The motion stimulus was then presented for 8.4 s, and the observer attended to the motion stimulus in the peripheral visual field. To keep attention on the stimulus, he or she counted the targets while fixating on the line presented in the center. Targets were presented in the annular area between concentric circles with diameters of 28.4 deg and 9.4 deg, as shown in Fig 6. Targets always appeared from one edge of the annular area and disappeared at the opposite edge. The velocity of targets was the same as that of stimulus dots. Stimulus and target dots were simultaneously presented from the beginning of each trial. The direction of the motion was vertical or horizontal and always opposite to that of the center. Observers responded with the number of targets counted after the presentation of the motion stimulus. The response time was unlimited and feedback was not provided to the observer. The next trial was launched by pressing a button after a response. The direction of motion in the peripheral area was one of four directions (left, right, up, or down) and was randomized for each trial. The trial of each direction was repeated five times for each observer.

In the “central attention” condition, observers attended to the central area in the stimulus. The procedures were identical as those in the condition of “peripheral attention,” except for the attended area and the area where targets were presented (the central area). This condition was conducted in separate sessions.

We also conducted the “no-attention” condition to clarify the effect of attention, in which observers were instructed not to attend to any particular area of the stimulus and keep eyes on the fixation line at the center of the stimulus. It was considered that observer’s attention was directed at the fixation line for most part under this condition. There were three kinds of stimulus in this condition. In the first stimulus, motion stimuli with opposite directions were presented in the peripheral and central areas, and the targets were presented in the annular part of the peripheral area, as in the condition of peripheral attention. The second stimulus was the same as the first stimulus, except that the targets were presented in the central area. This stimulus was used to investigate the effect of exogenous attention drawn by the bright targets themselves. The third stimulus was the same as the first and second stimuli, except that the targets were not presented. This stimulus was to reveal the effect of exogenous attention by the motion stimulus itself.

One author and seven volunteers (six males and two females, aged between 22 and 32 years) participated in this experiment; they had also participated in Experiment 1. The apparatuses for stimulus presentation and eye recording and the data analysis methods were the same as those used in Experiment 1.

### Results

**Fig 7:**
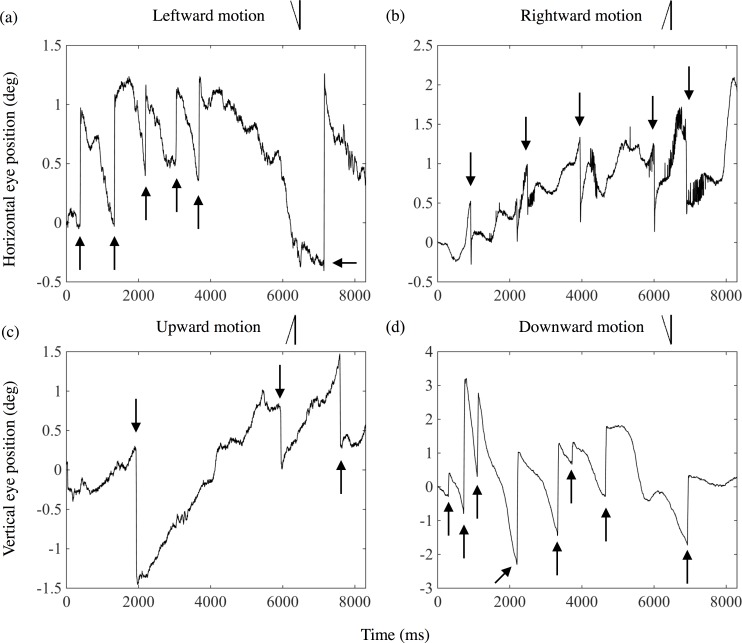
Representative trace of eye position in a trial for an observer (OB4) in Experiment 2. Four panels depict the results when attending to (a) leftward, (b) rightward, (c) upward, and (d) downward motions. Formats of the figures are the same as those in Fig 3.

A representative tracing of eye position for observer OB4 during one trial in the peripheral attention condition is shown in Fig 7. The panels present the results when attending to the leftward, rightward, upward, and downward motion, respectively. The horizontal axis shows the time [ms] from the onset of stimulus. The vertical axis shows the horizontal or vertical position of the eye, as in Fig 3. The line drawing inserted in the upper part of each panel presents the shape of predicted OKN corresponding to the direction of the attended motion for each motion condition, as in Fig 3.

In Fig 7A, it can be observed that OKN corresponding to the leftward motion occurred around 300, 1400, 2200, 3000, 3600 and 7000 ms (indicated by arrows). Similarly, in Fig 7B, OKN corresponding to the rightward motion occurred around 1000, 2300, 4000, 6000 and 7000 ms. In the data for Fig 7C and 7D, OKN corresponding to the upward and downward motion occurred 3 times and 8 times, respectively. In Fig 7B, a small saccade occurred immediately after each fast phase, but these are considered as corrective saccades and not used in this analysis.

**Fig 8:**
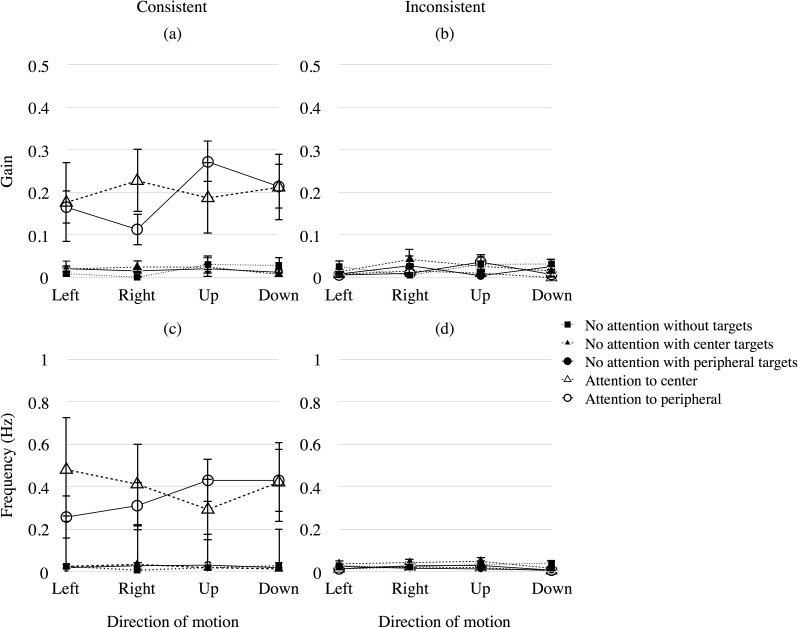
Mean gain and frequency of OKN in Experiment 2. The x-axis shows the gain ((a), (b)) and frequency ((c), (d)) of OKN. Right and left panels show the results of OKN with consistent and inconsistent directions to the targets (except in the no attention condition, in which the targets were not presented and consistent OKN indicates the OKN consistent with central motion). Error bars show ±1 SEM.

Fig 8 presents the mean gain ((a), (b)) and frequency ((c), (d)) of OKN for each combination of attention condition and direction of attended or the targets’ direction of motion across observers. The y-axis shows the gain ((a), (b)) and frequency ((c), (d)) of OKN. The x-axis shows the direction of motion. The circle, triangle, and square symbols respectively show the results of the different conditions of targets: targets in peripheral area, targets in the central area and no targets. The open and filled symbols show the results in the attention condition and those in the no-attention condition, respectively. The left panels ((a), (c)) present the results of OKN corresponding to the direction of targets. The right panels ((b), (d)) present the results of OKN not corresponding (opposite) to the direction of targets. The square in left panels indicates the results of OKN corresponding to the direction of central motion. Error bars show ±1 SEM.

The gain and frequency of OKN corresponding to the direction of the attended motion were relatively larger than those of OKN in other attention conditions regardless of the direction of motion (open symbols in Fig 8A and 8C). As in the results of Experiment 1, the gain and frequency of OKN not corresponding to the direction of attended motion were small (open symbols in Fig 8B and 8D). In addition, only a few OKN occurred when observers did not attend to the motion stimulus, even though the motion stimulus was presented in the center (filled symbols in Fig 8C and 8D). Although in comparison to the general results of OKN in the previous studies, it seems strange that the motion stimulus in the central visual field produced only few OKN in this experiment, the present result indicates that OKN occurred depending on the attended motion even though it is presented in the periphery and another motion is presented in the center. We believe that the motion in the attended area, rather than that in the central visual field, is important for the production of OKN, and that OKN produced by the motion in the central visual field is usually quite high because the motion in the center may draw visual attention quite strongly.

To verify the significance of the results mentioned above, a three-way ANOVA (*attention*, *direction of motion*, and *targets*) was performed on the consistent data for the four conditions (Attention to center, Attention to peripheral, No attention with peripheral targets, and No attention with center targets). The main effect of *attention* was significant for gain (*F*(1, 7) = 11.613, *p* < .05) and frequency of OKN (*F*(1, 7) = 6.988, *p* < .05). The main effect of the *direction of motion* was not significant for gain (*F*(3, 21) = 0.866, *p* > .10) and frequency of OKN (*F*(3, 21) = 0.250, *p* > .10). The main effect of *targets* was not significant for gain (*F*(1, 7) = 0.032, *p* > .10) and frequency of OKN (*F*(1, 7) = 0.094, *p* > .10). The interaction of the *direction of motion* versus *targets* was significant (*F*(3, 21) = 3.5708, *p* < .05) for gain of OKN. The interaction among *attention*, *direction of motion*, and *targets* was marginally significant (*F*(3, 21) = 2.516, *p* < .10) for gain of OKN. Multiple comparison tests using Ryan’s method (α = 0.05) in peripheral attention showed that the difference between the upward and leftward motion, the upward and rightward motion, and the downward and leftward motion differed significantly (*p* < .05). This trend is the same as in the results of frequency for direct condition in Experiment 1. The *attention* and *direction of motion* interaction and *targets* and *attention* interaction were not significant for gain and frequency of OKN (*p* > .10).

To confirm that observers did not directly look at the motion stimulus during the trials, the eye position was analyzed, as in Experiment 1. In the “peripheral attention” condition, the averages (and standard deviations) of the horizontal deviation for vertical motion was 0.16 (0.46) deg. The average of the vertical deviation for horizontal motion was 0.81 (1.33) deg. These results demonstrate that the observers did not directly look at the motion stimulus because the closest edge of the stimulus was 4.7 deg apart from the fixation line. Therefore, we conclude that OKN in Experiment 2 occurred not by looking directly at the motion stimulus but through visual attention.

## Discussion

### Effects of attention on OKN

By comparing the results of attention and no-attention conditions in Experiments 1 and 2, it is shown that attention to an area of motion produces OKN corresponding to the motion. These results extend the results of previous studies [5,6] showing that OKN can be facilitated by attention to peripheral motion in isolation. In Experiment 1, when observers attended to the peripheral motion while fixating on a central line, OKN occurred at 0.25 Hz and the gain of OKN was 0.13 on average, and when they did not attend to the motion of the same stimulus, OKN occurred at 0.02 Hz and the gain of OKN was 0.01 on average. The frequency and gain of OKN in the attention condition was significantly higher than that in the no-attention condition. We confirmed that the result in the attention condition was due to the attention to the motion stimulus.

In Experiment 2, we measured OKN using a stimulus having center and peripheral areas with opposite motions. When attending to the motion stimulus in the periphery, OKN consistent with the peripheral motion occurred, while OKN consistent with the motion stimulus with the opposite direction presented at the center did not occur. This result, however, is inconsistent with a previous study [14], which stated that OKN corresponding to the motion in the central visual field occurred even if the observer attended to the peripheral motion in an opposite direction to the central motion. We presume that the main reason for the difference is the existence of the attentional task. We suppose that attention was not strong enough to produce OKN in the previous study because observers were instructed to just attend to the peripheral motion without an attentional task. This view is consistent with a recent study showing that OKN gain increases when a moving feature in the peripheral visual field is the focus of attention while fixating on the central point [12]. In the present study, steady and focused attention was paid to the peripheral motion by an attentional task. A previous study claimed that binocularly fused motion inputs in the central visual field have priority for the oculomotor system over those in peripheral visual field with attention. However, the results of the present study indicate that motion information in the peripheral visual field may have priority for generating OKN if attention is paid to the field.

In Experiment 1, OKN gain was 0.13, on average, when observers attended to the peripheral motion, which was less than that of a previous study, in which the gain was about 0.25 when the stimulus speed was 15 deg/s and the size of the central deletion was 10 deg [5]. We presume that this difference is mainly due to the difference in the stimulus configurations. It is generally known that OKN gain decreases as the area of the moving display decreases [4] and when there are stationary edges in the moving stimulus [9]. The presentation area in Experiment 1 (34.1 × 10.2 or 13.0 × 28 deg) was smaller than that of the previous study (80 × 60 deg), and the fixation stimulus was presented in the present experiment; however, no fixation was presented in the previous study.

Exogenous attention [10] may be drawn by bright targets in the motion stimulus without endogenous attention in the no-attention condition in Experiments 1 and 2 because little OKN occurred in this condition. However, we suppose that the effect of exogenous attention by the targets and the motion stimulus itself for producing OKN was ignorable, at least for the case of the present experiment, from the results of the no-attention condition in Experiment 2. In that experiment, we used a stimulus having an opposite motion in concentric areas with three kinds of target arrangement with targets in the periphery, the center, and with no target. We found that the gain and frequency of OKN were not significantly different for three patterns of target presentation (Fig 8; No attention).

### Effects of stimulus motion in the central visual field on OKN

Surprisingly, the gain and frequency of OKN consistent with the direction of the attended motion in the peripheral visual field were nearly the same when motion stimulus in the opposite direction was presented in the center (gain = 0.19, frequency = 0.36 Hz, Experiment 2, Fig 8A and 8C; Attention to peripheral) and when no motion stimulus was presented in the center (gain = 0.13, frequency = 0.25 Hz, Experiment 1, Fig 4; Attention (consistent)). We compared these results statistically to verify the observation from the figures. A one-way ANOVA was performed on the averaged data for two conditions across experiments (the Attention condition in Experiment 1 and the Attention to peripheral condition in Experiment 2). The main effect of the condition was not significant for both gain and frequency of OKN (gain: *F*(1, 17) = 1.630, *p* > .10; frequency: *F*(1, 17) = 0.704, *p* > .10). Although it has been reported that motion in the central visual field is important to produce corresponding OKN [5–7], the present study’s results indicate that motion in the peripheral visual field with attention produced corresponding OKN. It has been reported that the non-disparate area of the display determines the direction of the pursuit phase of OKN when two different directions of motion with disparity differences were simultaneously presented at the central and peripheral visual field [14]. Thus, we presume that OKN consistent with the direction of attended motion occurred when the central and peripheral displays with different directions of motion were coplanar.

The difference in the area of motion in the periphery seemed to have some effect on the results in the conditions of peripheral attention in Experiments 1 and 2. This is because, when the stimulus area in Experiment 2 (899 deg^2^) was about 2.5 times as large as that in Experiment 1 (347.8 deg^2^ in the LR pattern and 369.2 deg^2^ in the UD pattern), the gain and frequency of OKN in Experiment 2 (gain = 0.19, frequency = 0.35) were about 1.4 times as large as that in Experiment 1 (gain = 0.13, frequency = 0.25). However, the stimulus area in a previous study (4800 deg^2^) [5] was about 10 times as large as that in Experiment 1, and the gain of OKN in the study [5] was about 1.9 times as high as that in Experiment 1. The comparison between the results in the studies shows that the gain of OKN and the area of motion stimulus could be related, although the effect of the stimulus area seems insufficient to explain the difference in the results in Experiments 1 and 2.

OKN was facilitated when the position of attention and that of the gaze were the same. The frequency of OKN was 2.98 Hz and gain of OKN was 0.82 on average, when observers looked at the motion stimulus in the central visual field directly with attention in Experiment 1 (Fig 5). There is definitely a strong effect of foveal presentation of motion on OKN, and this is plausible from the strong bias of this region in the retinal projection to the cortex. One of the reasons why the central stimulation produces OKN with high frequency and gain would be high contrast sensitivity in the fovea [19]. In addition, the magnitude of attention seems to differ between the central and peripheral attentions while fixating the center of stimulus because some attention would remain in the center for fixation in the case of peripheral attention. However, the possible difference in the magnitude of attention or in the contrast sensitivity for the cases of central and peripheral attention would be too small to produce different gain and frequency of OKN because the results of them for the two attention conditions in Experiment 2 are almost the same (Fig 8). The general way of interpreting the results in Experiment 1 (Fig 5) would be to note that OKN produced by the stimulation at the center is facilitated by attention at the area. However, another way of interpretation is that OKN produced by attention is facilitated by the gaze at the center. We suppose that the latter interpretation would be more plausible from the present results in Experiment 2 showing that even when a motion stimulus was presented in the central area, OKN corresponding to the opposite motion in the peripheral area occurred if attention was strong in the area.

### Direction asymmetry in OKN

It is known that vertical OKN is asymmetrical with an upward preference [20,21]. The result of this study that there is no difference between upward and downward OKN is inconsistent with results of previous studies. One of the reasons for the difference would be the condition of stimulus velocity. In most conditions, upward and downward OKNs are different in the properties, but close inspection shows that there is no difference between upward and downward OKNs when the velocity of stimulus is low, about 10 deg/s, in previous studies [20,21]. We presume that the asymmetry in vertical OKN was not found because the velocity of the present stimulus was relatively low, that was 13.3 deg/s.

The frequency and gain of OKN being consistent with vertical motion were significantly higher than those of horizontal motion in Experiment 1 for the direct attention condition and Experiment 2 for the peripheral attention condition, respectively. We presume that the difference between horizontal and vertical OKNs could be due to the difference in the stimulus area used in the present study.

### Distinction between OKN and smooth pursuit eye movements

Eye movements observed in this study may be a kind of smooth pursuit eye movements because the condition used in this study was similar to that of smoothly pursuing a target dot when there was a stationary background [22–24], assuming that fixation is a special case of zero-velocity smooth pursuit. Actually, it is difficult to distinguish between OKN and smooth pursuit eye movements. Occurrence of vection could be one of indexes for the distinction, but it did not occur in this study because stimulus size was not large enough. Even if the eye movements observed in this study were a little different from normal OKN, it is obvious that the gain and frequency of them were affected by attention.

We suppose, however, that the eye movements observed in the present study can be regarded as OKN because saccades (fast phases) occurred even though the eye position was at the center of the fixation line (e. g. the third arrow in Fig 3A, the second and third arrow in Fig 3D and the first and second arrow in Fig 7A). If eye movements observed in the attention condition were smooth pursuit, these saccades should not occur under that circumstance of foveal observation of object. Therefore, we suppose that the eye movements observed in the present study can be regarded as OKN to keep the image of the continuously moving stimulus.

## Conclusions

In Experiment 1, we investigated whether OKN consistent with the direction of an attended motion occurred when stimuli with opposite directions of motion were simultaneously presented in a peripheral visual field; the results showed that OKN consistent with the direction of the attended motion occurred. In Experiment 2, we investigated whether OKN consistent with the direction of attended motion stimulus occurred when stimuli with opposite directions of motion were presented at the center and periphery of the visual field simultaneously, and the results also showed that OKN consistent with the direction of attended motion occurred. Eye position analysis confirmed that OKN in the experiments occurred not by looking at the motion stimulus directly but through visual attention.

Recently, several reports have described visual attentions related to pupillary light reflex [25–27], microsaccades [28,29] and vergence eye movements [30,31]. We suppose that it is possible to estimate the position of attention based on OKN when there are objects or areas of motion in the visual field. For example, systems using OKN make it possible to estimate the location where a driver is attending, to the left or right in the optical flow, while driving. In addition, it is also possible to estimate the position and state of attention more accurately by combining knowledge from the findings in the present study and those of the studies described above. To develop a system to estimate the position and state of attention, we need to completely ascertain the spatial and temporal properties of attentional modulation in OKN.

## Supporting information

S1 Table Averaged data for each observer. (XLSX)

## References

[1] Carpenter RH. Eye movements. Florida: CRC Press; 1991.

[2] Barnes G, Crombie JW. The interaction of conflicting retinal motion stimuli in oculomotor control. Exp Brain Res. 1985; 59: 548–558. 4029327 10.1007/BF00261346

[3] Dichgans J. Optokinetic Nystagmus as dependent on the retinal periphery via the vestibular nucleus In: Baker R, Berthoz A, editors. Control of Gaze by Brain Stem Neurons, Developments in Neuroscience. North Holland: Elsevier; 1977 pp. 261–267.

[4] Cheng M, Outerbridge JS. Optokinetic nystagmus during selective retinal stimulation. Exp Brain Res. 1975; 23: 129–139. 1183500 10.1007/BF00235455

[5] Dubois MFW, Collewijin H. Optokinetic reactions in man elicited by localized retinal motion stimuli. Vis Res. 1979; 19: 1105–1115. 550568 10.1016/0042-6989(79)90005-1

[6] Gresty M, Halmagyi H. Following eye movements in the absence of central vision. Acta Oto-lar. 1979; 87: 477–483. 10.3109/00016487909126455 463520

[7] van Die G, Collewijn H. Optokinetic nystagmus in man. Role of central and peripheral retina and occurrence of asymmetries. Human Neurobiol. 1982; 1: 111–119. 7185785

[8] Abadi RV, Howard IP, Ohmi M, Lee EE. The effect of central and peripheral field stimulation on the rise time and gain of human optokinetic nystagmus. Perception. 2005; 34: 1015–1024. doi: 10.1068/p5251b 16178157 10.1068/p5251b

[9] Murasugi CM, Howard IP, Ohmi M. Optokinetic nystagmus: the effects of stationary edges, alone and in combination with central occlusion. Vis Res. 1986; 26: 1155–1162. 3798750 10.1016/0042-6989(86)90049-0

[10] O’Craven KM, Rosen BR, Kwong KK, Treisman A, Savoy RL. Voluntary attention modulates fMRI activity in human MT–MST. Neuron. 1997; 8: 591–598. 10.1016/s0896-6273(00)80300-1 9136768

[11] Williams IM, Mulhall L, Mattingley J, Lueck C, Abel L. Optokinetic nystagmus as an assessment of visual attention to divided stimuli. J Clin Neurosci. 2006; 13: 828–833. doi: 10.1016/j.jocn.2005.10.010 16935509 10.1016/j.jocn.2005.10.010

[12] Rubinstein NJ, Larry AA. Optokinetic nystagmus suppression as an index of the allocation of visual attention. Invest Ophthalmol Vis Sci. 2011; 52: 462–467. doi: 10.1167/iovs.10-6016 20811052 10.1167/iovs.10-6016

[13] Maruyama M, Kobayashi T, Katsura T, Kuriki S. Early behavior of optokinetic responses elicited by transparent motion stimuli during depth-based attention. Exp Brain Res. 2003; 151: 411–419. doi: 10.1007/s00221-003-1497-2 12811443 10.1007/s00221-003-1497-2

[14] Howard IP, Gonzalez EG. Human optokinetic nystagmus in response to moving binocularly disparate stimuli. Vis Res. 1987; 27: 1807–1816. 3445470 10.1016/0042-6989(87)90109-x

[15] Posner MI. Chronometric explorations of mind. Oxford: Lawrence Erlbaum; 197.

[16] Brainard DH. The psychophysics toolbox. Spatial Vis. 1997; 10: 433–436. 9176952

[17] Pelli DG. The VideoToolbox software for visual psychophysics: Transforming numbers into movies. Spatial Vis. 1997; 10: 437–442. 9176953

[18] Kleiner M, Brainard D, Pelli D, Ingling A, Murray R, Broussard C. What’s new in Psychtoolbox-3. Perception. 2007; 36: 14.

[19] Virsu V, Rovamo J. Visual resolution, contrast sensitivity, and the cortical magnification factor. Exp Brain Res. 1979; 37: 475–494. 520438 10.1007/BF00236818

[20] Van den Berg AV, Collewijn H. Directional asymmetries of human optokinetic nystagmus. Exp Brain Res. 1988; 70: 597–604. 3384058 10.1007/BF00247608

[21] Murasugi CM, Howard IP. Up-down asymmetry in human vertical optokinetic nystagmus and after nystagmus: contributions of the central and peripheral retinae. Exp Brain Res. 1989; 77: 183–192. 2792261 10.1007/BF00250580

[22] Murphy BJ, Kowler E, Steinman RM. Slow oculomotor control in the presence of moving backgrounds. Vis Res. 1975; 15: 1263–1268. 1198940 10.1016/0042-6989(75)90172-8

[23] Collewijn H, Tamminga EP. Human smooth and saccadic eye movements during voluntary pursuit of different target motions on different backgrounds. J Physiol. 1984; 351: 217–250. 6747865 10.1113/jphysiol.1984.sp015242 PMC1193114

[24] Kowler E, Van Der Steen J, Tamminga EP, Collewun H. Voluntary selection of the target for smooth eye movement in the presence of superimposed, full-field stationary and moving stimuli. Vis Res. 1984; 24: 1789–1798. 6534002 10.1016/0042-6989(84)90010-5

[25] Binda P, Pereverzeva M, Murray SO. Attention to bright surfaces enhances the pupillary light reflex. J Neurosci. 2013; 33: 2199–2204. doi: 10.1523/JNEUROSCI.3440-12.2013 23365255 10.1523/JNEUROSCI.3440-12.2013 PMC6619119

[26] Mathôt S, Van der Linden L, Grainger J, Vitu F. The pupillary light response reveals the focus of covert visual attention. PLoS ONE. 2013; 8: e78168 doi: 10.1371/journal.pone.0078168 24205144 10.1371/journal.pone.0078168 PMC3812139

[27] Naber M, Alvarez GA, Nakayama K. Tracking the allocation of attention using human pupillary oscillations. Front Psychol. 2013; 10.3389/fpsyg.2013.00919 PMC3857913 24368904

[28] Hafed ZM, Clark JJ. Microsaccades as an overt measure of covert attention shifts. Vis Res. 2002; 42: 2533–2545. 12445847 10.1016/s0042-6989(02)00263-8

[29] Engbert R, Kliegl R. Microsaccades uncover the orientation of covert attention. Vis Res. 2003; 43: 1035–1045. 12676246 10.1016/s0042-6989(03)00084-1

[30] Solé Puig M, Pérez Zapata L, Aznar-Casanova JA, Supèr H. A role of eye vergence in covert attention. PloS ONE. 2013; 8: e52955 doi: 10.1371/journal.pone.0052955 23382827 10.1371/journal.pone.0052955 PMC3561361

[31] Solé Puig M, Puigcerver L, Aznar-Casanova JA, Supèr H. Difference in visual processing assessed by eye vergence movements. PloS ONE. 2013; 8: e72041 doi: 10.1371/journal.pone.0072041 24069140 10.1371/journal.pone.0072041 PMC3777953

